# Neglschisandrins E–F: Two New Lignans and Related Cytotoxic Lignans from *Schisandra neglecta*

**DOI:** 10.3390/molecules18022297

**Published:** 2013-02-19

**Authors:** Min Chen, Xiumei Xu, Bing Xu, Panpan Yang, Zhihua Liao, Susan L. Morris-Natschke, Kuo-Hsiung Lee, Daofeng Chen

**Affiliations:** 1 Key Laboratory on Luminescence and Real-Time Analysis (Ministry of Education), College of Pharmaceutical Sciences, Southwest University, Chongqing 400715, China; 2 School of Life Sciences, Southwest University, Chongqing 400715, China; 3 Natural Products Research Laboratories, UNC Eshelman School of Pharmacy, University of North Carolina, Chapel Hill, NC 27599-7568, USA; 4 Chinese Medicine Research and Development Center, China Medical University and Hospital, Taichung, Taiwan; 5 Department of Pharmacognosy, School of Pharmacy, Fudan University, Shanghai 201203, China

**Keywords:** *Schisandra neglecta*, Schisandraceae, dibenzocyclooctadiene lignan, neglschisandrins E–F, cytotoxicity

## Abstract

Phytochemical investigation of an ethanolic extract of stems of *Schisandra neglecta* led to the isolation and identification of two new dibenzocyclooctadiene lignans, designated neglschisandrins E (**1**) and F (**2**), and thirteen known lignans. All structures and stereochemistries were determined by spectroscopic methods, including 2D-NMR techniques. The isolates were evaluated for *in vitro* cytotoxic activity. Among them, compounds **2**–**6** exhibited moderate to weak cytotoxicity against the human colorectal carcinoma HCT-8 cell line with EC_50_ values of 7.33~19.8 μg/mL. In addition, compounds **2**–**4** also exhibited marginal cytotoxicity against the human lung carcinoma A549 cell line with EC_50_ values of 11.8~15.0 μg/mL.

## 1. Introduction

Stems or fruits of plants in the Schisandraceae family are used widely in China as tonic and astringent agents for the treatment of rheumatic arthritis, traumatic injury, and related diseases [1]. Schisandraceae plants are rich in lignans, especially dibenzocyclooctadiene lignans, which show beneficial pharmacological effects, including anti-HIV, antitumor-promoting, calcium antagonistic, and anti-lipid peroxidative actions [2,3,4,5,6,7,8,9,10,11,12,13].

**Figure 1:**
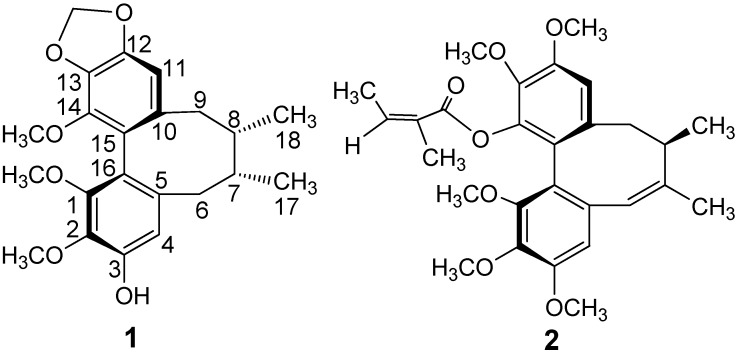
Structures of Compounds **1**–**2**.

In our previous study, we reported four new dibenzocyclooctadiene lignans from the stems of *Schisandra* *neglecta*, which is indigenous to the Tibet Autonomous Region of China [14,15]. Our further investigation of the same plant has now led to the isolation and identification of two new dibenzocyclooctadiene lignans, named neglschisandrins E (**1**) and F (**2**) (Figure 1), together with thirteen known lignans, 6-*O*-benzoylgomisin O (**3**) [16], (+)-γ-rubschisandrin (**4**) [17], rubschisantherin (**5**) [17], benzoylisogomisin O (**6**) [18], schisandrin A (**7**) [19], schisanhenol (**8**) [20], angeloylgomisin H (**9**) [21], gomisin H (**10**) [21], tigloylgomisin H (**11**) [21], benzoylgomisin H (**12**) [21], schisandrin (**13**) [22], gomisin B (**14**) [23], and angeloyl-(+)-gomisin K_3_ (**15**) [24]. This paper reports the isolation and structural elucidation of these compounds as well as their *in vitro* cytotoxicity against human lung carcinoma A549 and human colorectal carcinoma HCT-8 cell lines.

## 2. Results and Discussion

A 95% alcoholic extract prepared from stems of *Schisandra neglecta* was partitioned between diethyl ether and water. The diethyl ether extract was subjected to silica gel column chromatography (CC), preparative TLC, and semi-preparative reversed phase HPLC to give the two new and thirteen known dibenzocyclooctadiene lignans named above.

Compound **1**, obtained as amorphous powder, had the molecular formula C_22_H_26_O_6_ based on HR-ESI-MS (*m/z* 387.1810 [*M*+H]^+^). The UV and NMR spectra indicated that **1** was a dibenzocyclooctadiene lignan [25]. The ^1^H-NMR spectrum of **1** (Table 1) showed signals due to two secondary methyl groups (*δ*_H_ 0.97, 0.73, each 3H, *d*, *J* = 7.1 Hz), assignable to the *cis*-oriented Me-7 and Me-8, respectively [26]. The presence of four benzylic methylene signals (*δ*_H_ 2.25, 1H, *dd*, *J* = 13.0, 9.6; 2.01 Hz, 1H, *d*, *J* = 13.0 Hz; 2.55, 1H, *dd*, *J* = 13.6, 7.4; 2.45 Hz, 1H, *dd*, *J* = 13.6, 1.5 Hz) indicated that C-6 and C-9 were unsubstituted. The ^1^H-NMR spectrum also showed signals for two aromatic protons (*δ*_H_ 6.63, 6.49 each 1H, s), assignable to CH-4 and CH-11, respectively. Three methoxy groups [*δ*_H_ 3.50, 3.79 and 3.93, each 3H, s) and a methylenedioxy (–OCH_2_O–) moiety (*δ*_H_ 5.96, 5.95, each 1H, *d*, *J* = 1.3 Hz] were also observed as substituents on the aromatic rings. The proton signal at *δ*_H_ 5.68 (br s) showed no correlations in the HMQC spectrum, and was assigned as an hydroxyl on the aromatic ring. This assignment was supported by an IR band at 3447 cm^−1^ [27].

**Table 1:** ^1^H- (400 MHz) and ^13^C- (100 MHz) NMR data (measured in *CDCl_3_*) for compounds **1** and **2** (*δ* in ppm, *J* in Hz).

	1		2	
Position	*δ*_C_	*δ*_H_	*δ*_C_	*δ*_H_
			S	
1	150.3 (*s*)	-	152.3 (*s*)	-
2	137.4 (*s*)	-	139.9 (*s*)	-
3	148.8 (*s*)	-	152.6 (*s*)	-
4	110.2 (*d*)	6.63 (*s*)	106.3 (*d*)	6.38 (*s*)
5	140.3 (*s*)	-	136.5 (*s*)	-
6	35.1 (*t*)	2.25 (*dd*, *J* = 9.6,13.0)	125.3 (*t*)	6.20 (*s*)
		2.01 (*d*, *J* = 13.0)		-
7	40.9 (*d*)	1.79 (*m*)	142.2 (*s*)	-
8	33.8 (*d*)	1.88 (*m*)	34.2 (*d*)	2.79 (*m*)
9	38.9 (*t*)	2.55 (*dd*, *J* = 13.6,7.4)	42.9 (*t*)	3.10 (*dd*, *J* = 9,15.1)
		2.45 (*dd*, *J* = 13.6,1.5)		2.38 (*dd*, *J* = 10.0,15.1)
10	132.7 (*s*)	-	135.3 (*s*)	-
11	106.2 (*d*)	6.49 (*s*)	111.5 (*d*)	6.57 (*s*)
12	147.7 (*s*)	-	151.6 (*s*)	-
13	135.1 (*s*)	-	139.3 (*s*)	-
14	141.3 (*s*)	-	142.5 (*s*)	-
15	122.5 (*s*)	-	124.1 (*s*)	-
16	121.3 (*s*)	-	122.1 (*s*)	-
17	21.8 (*q*)	0.97 (*d*, *J* = 7.1)	18.9 (*q*)	1.63 (*s*)
18	12.4 (*q*)	0.73 (*d*, *J* = 7.1)	19.4 (*q*)	1.03 (*d*, *J* = 6.9)
*M*eO-C (1)	60.1 (*q*)	3.50 (*s*)	60.7 (*q*)	3.61 (*s*)
*M*eO-C (2)	61.0 (*q*)	3.93 (*s*)	60.8 (*q*)	3.84 (*s*)
*M*eO-C (3)	-	-	55.9 (*q*)	3.83 (*s*)
*M*eO-C (12)	-	-	55.9 (*q*)	3.88 (*s*)
*M*eO-C (13)	-	-	60.7 (*q*)	3.80 (*s*)
*M*eO-C (14)	59.7 (*q*)	3.79 (*s*)	-	-
HO-C (3)		5.68 (*brs*)		-
OCH_2_O	100.8 (*t*)	5.96, 5.95 (*d*, *J* = 1.3)	-	-
Ang:1'	-	-	165.2 (*s*)	-
2'	-	-	127.8 (*s*)	-
3'	-	-	136.8 (*d*)	5.86 (*m*)
4'	-	-	15.3 (*q*)	1.78 (*s*)
5'	-	-	20.3 (*q*)	1.72 (*d*, *J* = 7.1)

HMBC correlations of CH-11 (*δ*_H_ 6.49) and methylenedioxy (*δ*_H_ 5.96, 5.95) protons with aromatic carbons at *δ*_C_ 147.7, 135.1 indicated that the methylenedioxy group was located at C-12 and C-13. Correlations of the CH-4 proton (*δ*_H_ 6.63) with carbons at *δ*_C_ 148.8 (C-3) and 137.4 (C-2) and of the hydroxyl proton at *δ*_H_ 5.68 with carbons at *δ*_C_ 110.2 (C-4), 148.8 (C-3) and 137.4 (C-2), indicated that the hydroxyl was located at C-3. Thus, the three methoxy groups were located at C-1, C-2, and C-14, based on HMBC correlations of the protons at *δ*_H_ 3.79, 3.93 and 3.50 with aromatic carbons at *δ*_C_ 141.3 (C-14), 137.4 (C-2) and 150.3 (C-1), respectively.

**Figure 2:**
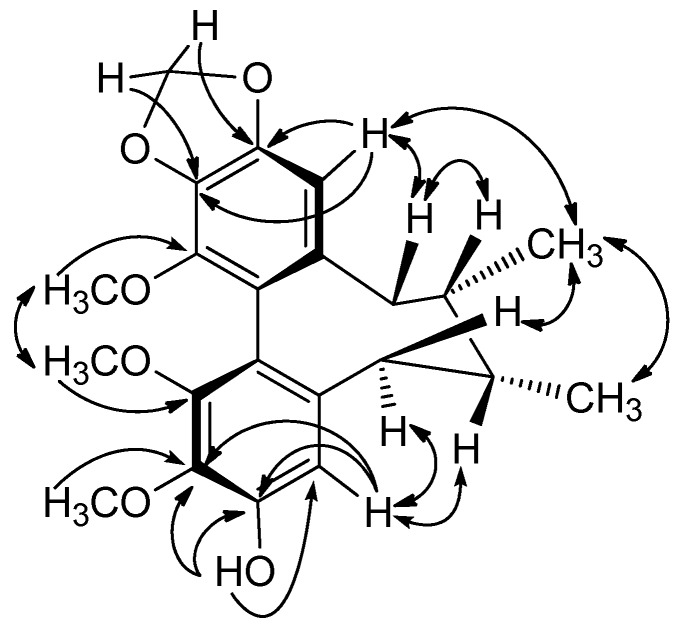
Key HMBC (→) and NOESY (↔) Correlations of **1**.

The circular dichroism (CD) spectrum of **1** showed a negative Cotton effect at 216 nm and a positive *Cotton* effect at 254 nm, which indicated an *R*-biphenyl configuration in **1** [28]. NOESY correlations between CH-11/Me-18, CH-4/CH-6α and CH-11/CH-9β indicated a twist-boat-chair (TBC) conformation for the cyclooctadiene ring [29] (Figure 2). The substituent positions and stereochemical assignments in the cyclooctadiene ring of **1** were further supported by NOESY correlations of MeO-1/MeO-14, Me-17/Me-18, CH-6β/Me-18 and CH-4/CH-7. Thus, the structure of **1** was determined as that shown in Figure 1. Compound **1** and (−)-gomisin L_2_ have the same planar structure, but different stereochemistry [30].

Compound **2**, obtained as colorless powder, has the molecular formula C_28_H_34_O_7_ according to HR-ESI-MS (*m/z* 505.2187 [*M*+Na]^+^). Its IR, UV, CD and NMR data indicated that **2** is a also a dibenzocyclooctadiene lignan. The ^1^H-NMR spectrum of **2** (Table 1) showed the presence of five methoxy groups at *δ*_H_ 3.61, 3.80, 3.83, 3.84 and 3.88 (each 3H, s), and an angeloyl group at *δ*_H_ 5.86 (1H, m), 1.78 (3H, s), 1.72 (3H, *d*, *J* = 7.1 Hz) on aromatic rings [24].

**Figure 3:**
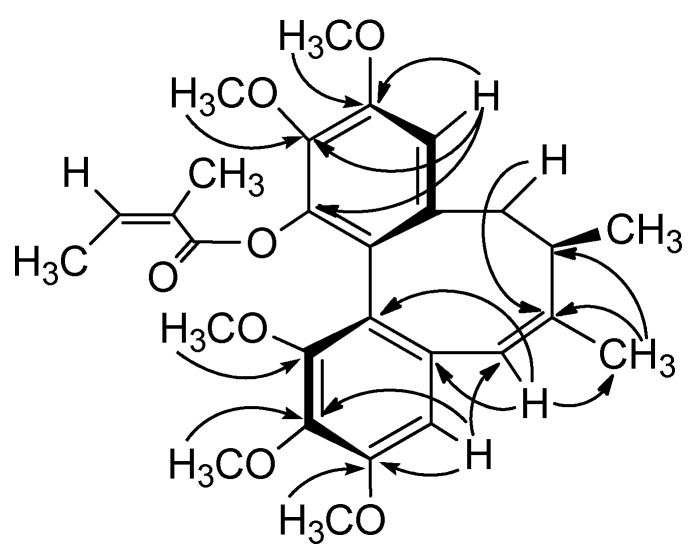
Key HMBC correlations of **2**.

The following proton-carbon HMBC correlations were observed: CH-4 (*δ*_H_ 6.38) with C-3 (*δ*_C_ 152.6) and C-2 (*δ*_C_ 139.9), CH-11 (*δ*_H_ 6.57) with C-12 (*δ*_C_ 151.6), C-13 (*δ*_C_ 139.3) and C-14 (*δ*_C_ 142.5), and the five methoxy signals at *δ*_H_ 3.61, 3.84, 3.83, 3.88 and 3.80 with aromatic carbon signals at *δ*_C_ 152.3 (C-1), 139.9 (C-2), 152.6 (C-3), 151.6 (C-12) and 139.3 (C-13), respectively. These correlations indicated that the five methoxy groups were attached to C-1, C-2, C-3, C-12, and C-13, and the angeloyl group was located at C-14 (Figure 3). 

Furthermore, the ^1^H-NMR spectrum of **2** showed a proton signal at *δ*_H_ 6.20 (1H, *s*) and the ^13^C-NMR spectrum showed a quaternary olefinic carbon at *δ*_C_ 142.2 and a methine olefinic carbon at *δ*_C_ 125.3, suggesting the formation of a double bond. A singlet methyl signal at *δ*_H_ 1.63 (3H, *s*, Me-17) in the ^1^H-NMR spectrum indicated that the double bond was located between C-6 and C-7 and was substituted with a methyl group. HMBC correlations of *δ*_H_ 6.38 (CH-4) with *δ*_C_ 125.3 (C-6), *δ*_H_ 6.20 (CH-6) with *δ*_C_ 136.5 (C-5), 122.1 (C-16) and 18.9 (C-17), *δ*_H_1.63 (Me-17) with *δ*_C_ 142.2 (C-7) and 34.2 (C-8), *δ*_H_ 2.38 and 3.10 (CH_2_-9) with *δ*_C_ 142.2 (C-7) confirmed the substructure.

**Figure 4:**
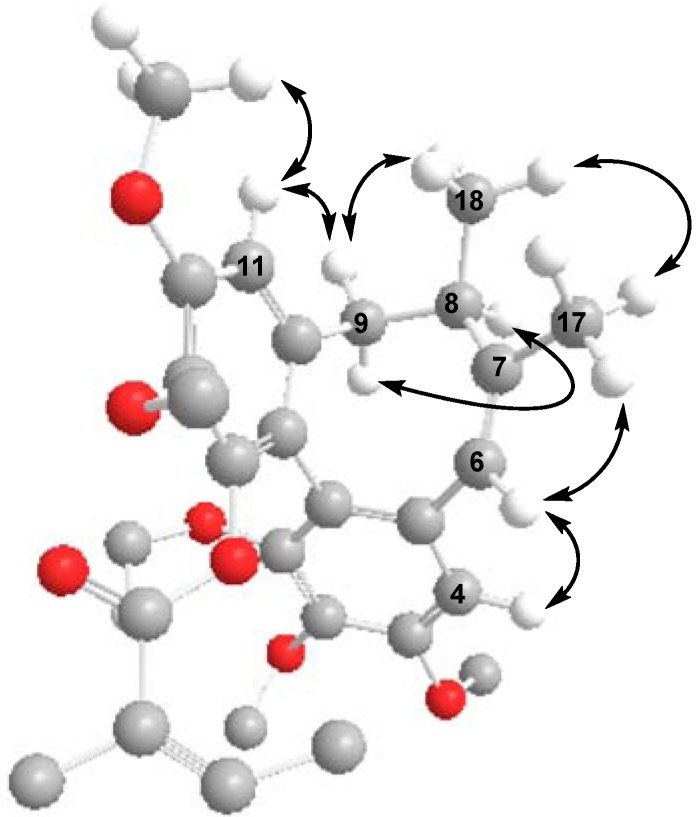
Key NOESY correlations of **2**.

The CD spectrum of **2** had a negative *Cotton* effect at 219 nm and a positive *Cotton* effect at 251 nm, indicating that **2** has an *R*-biphenyl configuration. The NOESY correlations between CH-4/CH-6, CH-6/Me-17, and CH-11/CH-9β, CH-9α/CH-8, CH-11/MeO-12, CH-9β/Me-18, and Me-17/Me-18 fully supported the assigned structure and stereochemistry (Figure 4). Compound **2** has an endocylic double bond rather than the exocyclic double bond found in the previously reported neglschisandrin B [14].

The lignans were evaluated *in vitro* for cytotoxicity against human lung carcinoma A549 and human colorectal carcinoma HCT-8 cell lines employing a MTT-assay with paclitaxel as the positive control (Table 2). Five lignans exhibited moderate cytotoxicity, while the remaining lignans showed no activity. Against HCT-8, compounds **3** and **6** showed moderate cytotoxicity (EC_50_ 9.58 and 7.33 μg/mL, respectively); compounds **2** and **4** exhibited marginal cytotoxicity (EC_50_ 13.8 and 12.6 μg/mL, respectively), and compound **5** showed weak cytotoxicity (EC_50_ 19.6 μg/mL). Against A549, compounds **2**–**4** exhibited marginal cytotoxicity with EC_50_ values ranging from 11.8 to 15.0 μg/mL. 

**Table 2:** Cytotoxicity data of lignans from *Schisandra neglecta.*

Compound	Cytotoxicity (EC_50_, μg/mL)	
	HCT-8	A549
**2**	13.8	11.8
**3**	9.58	13.8
**4**	12.6	15.09
**5**	19.8	NA ^a^
**6**	7.33	NA ^a^
Paclitaxel	0.21	<0.005

^a^ NA (not active)--test compound (20 μg/mL) did not reach 50% inhibition.

## 3. Experimental

### 3.1. General

Optical rotations: *P-1020* digital spectropolarimeter (JASCO) with MeOH as solvent. UV spectra: *Hitachi* *U-3010* spectrophotometer in MeOH; λ_max_ (log *ε*) in nm. ^1^H-, ^13^C-, 2D-NMR spectra: *Bruker DRX400* (400 MHz for ^1^H, 100 MHz for ^13^C) spectrometer in *CDCl_3_*; *δ* in ppm rel. to Me_4_Si as internal references. *J* in Hz. ESI mass: *Bio TOF Q* spectrometer; in *m/z* (rel. %). HR-ESI-MS: *Bruker Daltonics BioToF-ШQ* mass spectrometer in positive-ion mode (Bremen, Germany). IR spectra: *Avatar Thermo Nicolet* *360-ESP* spectrophotometer. TLC was performed on silica gel plates GF_254_ (Yantai Institute of Chemical Technology, Yantai, China). The TLC spots were visualized by UV light (254 nm) and sprayed with 10% H_2_SO_4_, followed by heating. Column chromatography (CC) was carried out on silica gel (200–300 mesh or 300–400 mesh Qingdao Marine Chemical Factory, Qingdao, China). Semi-prep HPLC was carried out on an octadecylsilane column (*RP-18*, 250 × 10 mm, 10 μm, YMC, detector: *Amersham UV-900*) with a flow rate of 3.0 mL/min.

### 3.2. Plant Material

Stems of *Schisandra neglecta* were collected in Lin-zhi County, Tibet Autonomous Region, People’s Republic of China in September of 2004, and identified by Professor Hong-ping Deng, School of Life Sciences, Southwest University. A voucher specimen (MC-LZ-040901) is deposited in the Herbarium of Medicinal Plant, School of Life Sciences, Southwest University, Chongqing, China.

### 3.3. Extraction and Isolation

Powdered air-dried stems (5.0 kg) of *Schisandra neglecta* were extracted exhaustively with 95% EtOH (5 × 10 L, each three days) at room temperature. The alcoholic extract was evaporated *in vacuo* to yield a semisolid (430 g), which was suspended in water (1,000 mL) and extracted five times with diethyl ether. The organic solution was concentrated to yield 112 g of residue, which was subjected to silica gel CC eluted with petroleum ether/acetone mixtures of increasing polarity (99:1 to 3:7) to obtain ten fractions. Fraction 3 (8.3 g), eluted with petroleum ether–acetone (95:5), gave **7** (1.2 g), and then was further chromatographed with silica gel CC eluting with petroleum ether/CHCl_3_ (9:2) to obtain six subfractions. Subfraction 3–3 (1.3 g) was subjected to preparative TLC with petroleum ether/CHCl_3_ (1:1) to yield **4** (14 mg). Fraction 4 (7.4 g), eluted with petroleum ether/acetone (9:1), was subjected to CC with petroleum ether/EtOAc (15:1~4:1) to obtain eight subfractions. Subfraction 4–3 (0.5 g) was purified by preparative HPLC with MeOH/H_2_O (70:30) to yield **5** (4 mg, RT 25.3 min) and **6** (10 mg, RT 31.1 min). Subfraction 4–4 (0.7 g) was subjected to preparative TLC with petroleum ether/CHCl_3_ (1:1) to yield **3** (17 mg). Subfraction 4–5 (0.3 g) was purified by semi-preparative HPLC with MeOH-H_2_O (70:30) to yield **1** (2 mg, RT 33.4 min). Subfraction 4–6 (3.2 g) was subjected to silica gel CC with petroleum ether-EtOAc (5:1) to yield **8** (2.3 g). Fraction 5 (6.7 g) eluted with petroleum ether-acetone (8:2) was subjected to silica gel CC with petroleum ether/EtOAc (10:2–5:2). Subfraction 5–2 (0.6 g) was purified by semi-preparative HPLC with MeOH/H_2_O (75:25) to yield **9** (32 mg, RT 37.5) and **15** (46 mg, RT 40.2). Subfraction 5–3 (1.7 g) was subjected to silica gel CC with petroleum ether/EtOAc (9:2), then purified by semi-preparative HPLC with MeOH/H_2_O (64:36–80:20) to yield **10** (4 mg, RT 25.6 min), **11** (10 mg, RT, 28.7 min), and **12** (43 mg, RT 37.9 min), Subfraction 5–5 (0.9 g) was subjected to silica gel CC with petroleum ether/CHCl_3_ (5:6), and further purified by semi-preparative HPLC with MeOH/H_2_O (7:3) to yield **13** (24 mg, RT 40.3 min) and **14** (19 mg, RT 44.5 min). Fraction 6 (5.2 g) eluted with petroleum ether-acetone (7:3) was subjected to silica gel CC with petroleum ether/EtOAc (10:3). Subfraction 6–3 (0.7 g) was purified by semi-preparative HPLC with MeOH-H_2_O (75:25 to 85:15) to yield **2** (10 mg, RT 24. 7 min).

*6R,7S,R-biar-[5,6,7,8-Tetrahydro-1,2,13-trimethoxy-6,7-dimethyl-benzo-[3',4']cycloocta[1',2':4,5]-benzo[1,2-d][1,3]dioxol-3-ol* (neglschisandrin E, **1**). White amorphous powder; [α]^22^_D_ +26.9° (c = 0.26, MeOH). UV (MeOH): 216 (4.67), 250 (4.22), 280 (3.97). CD (c = 0.08, MeOH), [θ]^15^ (nm): −28275 (216), +28636 (254). IR (KBr): 3447, 2925, 1615, 1585, 1476, 737. ^1^H-NMR and ^13^C-NMR: see Table 1. HR-ESI-MS: found 387.1810 ([M+H]^+^, C_22_H_2__7_O_6_^+^, calc. 387.1808).

*5Z,7R,R-biar-7,8-Dihydro-1,2,3,10,11-pentamethoxy-13-(2Z)-methylbut-2-enoyl-6,7-dimethyl-dibenzo[a,c]cycloocten-5(6H)-one* (neglschisandrin F, **2**). White amorphous powder; [α]^22^_D_ +16.9° (c = 0.31, MeOH). UV (MeOH): 216 (4.78), 250 (4.37), 279 (3.97). CD (c = 0.052, MeOH), [θ]^15^ (nm): −295132 (219), +155445 (251). IR (KBr): 3404, 2936, 1737, 1636, 1591, 1488, 734. ^1^H-NMR and ^13^C-NMR: see Table 1. HR-ESI-MS: found 505.2187 ([M+Na]^+^, C_28_H_34_NaO_7_^+^, calc. 505.2197).

### 3.4. Cytotoxicity Assay

Freshly trypsinized cell suspensions were seeded in 96-well microtiter plates at densities of 5,000–10,000 cells per well with compounds added from DMSO-diluted stock. After three days in culture, attached cells were incubated with cold 10% trichloroacetic acid (MTT, 0.5 mg/mL, 2 h) and subsequently solubilized in DMSO, and the isolates were tested against A549 and HCT-8 cancer cell lines using established colorimetric MTT assay protocols [31]. Paclitaxel was used as a positive control. All stock cultures were grown in T-25 flasks. The mean EC_50_ is the concentration of agent that reduces cell growth by 50% under the experimental conditions and is the average from at least three independent determinations that were reproducible and statistically significant. The absorbance was measured at 550 nm using a microplate reader.

## 4. Conclusions

Two new lignans, neglschisandrins E and F (**1** and **2**, respectively), and thirteen known lignans were isolated from stems of *Schisandra neglecta*. Their structures and stereochemistries were established by means of NMR and ESI-MS analyses, including 2D-NMR techniques. The isolates were evaluated for *in vitro* cytotoxic activity. Compounds **2**–**6** showed cytotoxicity against the human HCT-8 colorectal carcinoma cell line with EC_50_ values of 7.33~19.8 μg/mL, while compounds **2**–**4** also exhibited cytotoxicity against the human A549 lung carcinoma cell line with EC_50_ values of 11.8~15.0 μg/mL, respectively. Since other dibenzocyclooctadiene lignans in the genus *Schisandra* exhibit proven antitumor activities [32], further research to identify related potent cytotoxic compounds or explore detailed structure-activity relationships is merited.
